# Dataset for assessing the professional ethics of teaching by medical teachers from the perspective of students in Kermanshah University of Medical Sciences, Iran (2017)

**DOI:** 10.1016/j.dib.2018.09.060

**Published:** 2018-09-25

**Authors:** Yahya Safari, Nasrin Yoosefpour

**Affiliations:** Research Center for Environmental Determinants of Health (RCEDH), Kermanshah University of Medical Sciences, Kermanshah, Iran

**Keywords:** Professional ethic, Teaching ethic, Medical training, Medical ethic

## Abstract

The present data article was prepared with the aim of assessing the instructor׳s professional ethics of teaching in Kermanshah Medical Science University from the student׳s perspective in 2017. For this data article, 260 students in Kermanshah University of Medical Sciences (KUMS) were selected by a simple random sampling method. The data collection tool was based on “Ethical principles for college and university teaching” (Murray et al., 1996), teaching professional ethics questionnaire. The obtained data showed that the teachers have an average of (3.74 ± 0.73) in terms of personality characteristic, (3.48 ± 0.75) for dominating on content, (3.64 ± 0.64) in terms of dominating on teaching practices, (3.65 ± 0.63) for understanding the different learner׳s aspects, (3.65 ± 0.71) in terms of teaching assessment and 4.41 ± 0.78 for observing the educational regulations. These evaluated data were higher than the average level. The acquired data have shown that the instructors teaching professional ethic were higher than the average level, but still it was not ideal. Therefore, preparing and editing the teaching professional ethics charter and putting it in educational content during the teacher׳s service are suggested for the promotion of this status.

**Specifications table**

**Table:** 

Subject area	Social sciences
More specific subject area	Health sciences
Type of data	Tables
How data was acquired	260 students in Kermanshah University of Medical Sciences (KUMS) were selected by a simple random sampling method. The data collected tool was based on [16], a teaching professional ethics questionnaire.
Data format	Raw, analyzed
Experimental factors	For collecting the data, the teaching professional ethics questionnaire in [16] was used.
Experimental features	The raw data analyzed by applying SPSS (Ver.21) software.
Data source location	Kermanshah, Iran
Data accessibility	Data are included in this article

**Value of the data**

• Besides the important factors related to the students (including lifestyle, quality of life, mental health, quality sleep, spiritual health, happiness, etc.) [1], [2], [3], [4], [5], [6], faculty members of universities play an important role in student learning. The obtained data can be used to discuss the professional ethics of teaching by medical teachers.
• In the university׳s system, the effectiveness of teaching process as an important field depends on a complex of individual and organizational factors [7]. The data here discuss some of these factors.
• The university׳s performance has a direct and indirect effect on society. Therefore, the data can be used to pay attention to the university׳s performance, particularly to see how professional ethics is necessary [8], [9], [10], [11], [12], [13], [14], [15].
• This is the first study in Kermanshah, and its data can be useful for decision making. In addition, the present study data can be useful for future similar studies in other locations of Iran.

## 1 Data

In this data article, the instructors teaching professional ethics have evaluated the perspective of 260 students. The explanatory analysis of the obtained data showed that the students have evaluated all of the teachers teaching professional ethical aspect higher than the average level. These aspects have included personal characteristics (71.03 ± 5.65), dominating the content (24.73 ± 2.75), dominating the teaching method (18.20 ± 1.95), recognizing the various aspects of learners (18.28 ± 2.06), standard evaluation (25.58 ± 3.25) and observing the educational regulation (22.08 ± 2.85). Based on Table 1, in the personal characteristic component, the effort for good manners with students with an average of 4.54 was the highest – not using the students for carrying out personal work had the least average, with 2.32.

**Table 1:** The perspective of students for personal characteristic aspects of teacher׳s professional ethic component.

Main component	Dimensions	Mean ± SD
Personality characteristics	Pay attention to your apparent dress	3.56 ± 0.72
	Having good human relationships	3.81 ± 0.58
	Practical commitment to religious values	3.83 ± 0.65
	Being together feedback with patience	3.70 ± 0.63
	Responsibility towards students	3.75 ± 0.62
	Avoid bad jokes	3.90 ± 0.64
	Avoid rebuking and blaming	3.89 ± 0.61
	Having enough motivation to teach	3.80 ± 0.57
	Acceptance of criticism against student statements	3.70 ± 0.74
	Not having gender discrimination in dealing with students	3.65 ± 0.70
	Do not question the performance of other professors	2.73 ± 1.39
	Welcome to collective decision making	3.78 ± 0.64
	Commitment to the secrecy of the students	3.85 ± .61
	Having insight and insight about the behavior of students	4.29 ± 0.80
	Not using students to do personal things	2.32 ± 1.34
	Attention to trusteeship and public property in the use of educational tools	4.29 ± 0.80
	Attempt to good mood	4.54 ± 0.64
	Having good self-esteem	3.96 ± 0.55
	Access at non-teaching hours	3.78 ± 0.64

Based on the Table 2, the data analysis has shown that in the dominating on content aspect the component of using the research evidence for explaining the theory position with the average of 3.86 was the highest and the teaching subject component without personal choices with the average of 2.75 was the lowest.

**Table 2:** The perspective of students about dominating on content aspect from teacher׳s professional ethic component.

**Main component**	**Dimensions**	**Mean ± SD**
Mastery of content	Use of research evidence to explain theoretical positions	3.86 ± 0.67
	Teaching topics without personal choices	2.57 ± 1.23
	Provide appropriate horizontal link between different topics	3.41 ± 0.77
	Provide an appropriate vertical connection between different topics	3.45 ± 0.66
	Dominate analysis and content selection methods	3.76 ± 0.64
	Assignment to subject and content	3.82 ± 0.49

Also, the data analysis in Table 3 on dominating the teaching method aspect has shown that using various teaching method component with the average of 3.75 has the highest – proper organizing of lessons subject in lesson-term design with the average of 3.58 has the lowest.

**Table 3:** The perspective of students about dominating on teaching method aspect from the teacher׳s professional ethic component.

**Main component**	**Dimensions**	**Mean ± SD**
Mastery of teaching practice	Using of teaching methods variety	3.72 ± 0.66
	Presenting content by a continuous and conceptual way	3.64 ± 0.6
	Attention to student participation	3.61 ± 0.63
	Appropriate organizing of courses in the form of a curriculum- semester	3.58 ± 0.66
	Using of teaching methods in a consonant manner with goals and content	3.63 ± 0.63

For recognizing various aspects of learners, Table 4 has shown that the being skillful component has the average of 3.69, having the highest average. Trying to recognize student׳s previous experiences and knowledge, with the average of 3.59, has the least effect in creating motivation for students.

**Table 4:** The perspective of students about recognizing various aspect of learner from teacher׳s professional ethic aspect.

**Main component**	**Dimensions**	**Mean ± SD**
Identify the different learner aspect	Pay attention to individual differences of students	3.66 ± 0.57
	Identify the needs of students	3.66 ± 0.63
	An attempt to identify previous students׳ experiences and lessons learned	3.59 ± 0.69
	Being skilled in motivating	3.69 ± 0.62
	Strengthening the balance between cognitive, emotional and functional areas of learners	3.66 ± 0.63

Table 5 is about standard evaluation and has shown that determining a time component for checking the test papers and probable protest with the average of 3.91 was the highest, and determining assessing method from past with 3.48 was the lowest.

**Table 5:** The perspective of students about teachers׳ evaluation aspect from teachers׳ professional ethic aspect.

**Main component**	**Dimensions**	**Mean ± SD**
Standard evaluation	Favorable attention to feedback from students	3.66 ± 0.68
	Evaluation in line with educational goals	3.60 ± 0.65
	Observance of justice in evaluation	3.57 ± 0.7
	Determination of the evaluation method before evaluation	3.48 ± 0.84
	Use the appropriate incentive system to manage students׳ behavior	3.64 ± 0.68
	The use of formative evaluation	3.70 ± 0.66
	Determine the time to check the exam sheet and possible objections	3.91 ± 0.75

Evaluating Table 6 has shown that for observing the educational regulation aspect being committed for university regulation component with 4.56 have the highest average, and being on time in class with 4.13 has the lowest average.

**Table 6:** The perspective of students for observing the educational regulation aspect from teacher׳s professional ethic aspect.

**Main component**	**Dimensions**	**Mean ± SD**
**Observe educational rules**	Timely presence at the class	4.13 ± 0.83
	Pay attention to the attendance	4.41 ± 0.82
	Observe the legal period of the class	4.45 ± 0.89
	Match the assigned assignments with educational objectives	4.51 ± 0.72
	Commitment to university regulations	4.56 ± 0.63

## 2 Experimental design, materials and methods

The statistical population includes the students of Kermanshah University Medical Sciences in 2017. The sampling was carried out with simple random sampling and 260 people were selected as statistical a sample. For collecting data, the Murray et al. (1996) teaching professional ethic questionnaire was used [16]. The main questionnaire includes 44 locutions and 9 aspects which were validated by Sobhani Nejad et al. (2015) in Iran society [17]. The questionnaire have 48 locution and 6 component, personal characteristic, dominating on content, dominating on teaching method, recognizing the different aspects of learners, standard evaluation and observing the educational designing regulations. The answers were designed based on the 5 choices Likert range, from very low (=1) to very high (=5) and the scores was from very low (=1) to very high (=5). Araste et al. (2015) have reported that in Iranian samples for this questionnaire the total reliability coefficient and sub-scale were higher than 0.9 [18].
